# Actinomycosis of the Lower Jaw

**Published:** 1896-11

**Authors:** 


### ABSTRACTS FROM FOREIGN JOURNALS.

UNDER THE DIRECTION OF

J. PRESTON HOSKINS, PRINCETON, N. J.

ACTINOMYCOSIS OF THE LOWER JAW. Ducor, of Paris, reports a case of an enormous tumor on the lower jaw, with great emaciation and general distress. The patient belonged to the upper classes and submitted to treatment from twenty surgeons during eight years before a correct diagnosis and relief were obtained. Ducor suspected and established the presence of the ray fungus, and secured great improvement with potassium iodide, 2.5 grammes per day, painting the intrabuccal surface of the tumor with tincture of iodine, and injecting it into the parenchyma, mixed with equal parts of glycerin. Potassium iodide in this case again showed itself the specific remedy for actinomycosis, although the lesions were of too long standing to expect complete recovery.—*Monthly Retrospect of Medicine and Pharmacy*.

